# Targeted Downregulation of MYC through G-quadruplex Stabilization by DNAi

**DOI:** 10.3390/molecules26185542

**Published:** 2021-09-13

**Authors:** Alexandra Maria Psaras, Katarina T. Chang, Taisen Hao, Tracy A. Brooks

**Affiliations:** 1 Department of Pharmaceutical Sciences, School of Pharmacy and Pharmaceutical Sciences, Binghamton University, Binghamton, NY 13902, USA; apsaras@binghamton.edu (A.M.P.); ktchang@som.umaryland.edu (K.T.C.); 2 Department of BioMolecular Sciences, School of Pharmacy, University of Mississippi, University, MS 38677, USA; thao@coh.org

**Keywords:** MYC, G-quadruplex, transcriptional regulation, DNAi

## Abstract

Modulating the expression or function of the enigmatic MYC protein has demonstrated efficacy in an array of cancer types and a marked potential therapeutic index and safety profile. Despite its high therapeutic value, specific and selective inhibitors or downregulating therapeutics have proven difficult to develop. In the current study, we expanded our work on a MYC promoter G-quadruplex (G4) stabilizing DNA clamp to develop an oligonucleotide interfering DNA (DNAi) therapeutic. We explored six DNAi for G4-stabilization through EMSA, DMS footprinting, and thermal stability studies, focusing on the DNAi 5T as the lead therapeutic. 5T, but not its scramble control 5Tscr, was then shown to enter the nucleus, modulate cell viability, and decrease MYC expression through G4-stabilization. DNAi 5T is thus described to be our lead DNAi, targeting MYC regulation through stabilization of the higher-order DNA G4 structure in the proximal promoter, and it is poised for further preclinical development as an anticancer therapeutic.

## 1. Introduction

MYC, a basic helix-loop-helix/leucine zipper transcription factor, affects cellular proliferation, apoptosis, metastasis, angiogenesis, and microenvironment regulation [1]. Dysregulated MYC has been noted in many disorders [2,3,4,5,6,7,8,9,10,11,12], but it is first and foremost known for its oncogenic role [13,14,15,16] and in particular for its involvement in lymphomagenesis [17,18,19]. MYC is rearranged, amplified, or otherwise overexpressed in multiple types of non-Hodgkin’s lymphoma, including ~100% of Burkitt’s (BL) [20], 30% of diffuse large B-cell (DLBCL) [21,22,23], and >50% of mantle cell (MCL) lymphomas [24]. Translocations involving the MYC gene define BL, change in MYC is a central molecular characteristic underlying a genetic, versus phenotypic, characterization of DLBCL, wherein increased MYC activity is a negative prognostic factor, and MYC overexpression is correlated with decreased survival in MCL. Increased MYC expression or activity in both DLBCL and MCL correlates with a poor response to the standard chemotherapeutic regimen, CHOP [22,24,25,26,27,28]. Approaches that result in decreased MYC expression have been shown to significantly impact tumor viability while offering a notable therapeutic window and overall safe profile [29,30,31].

As we and other groups have shown, stabilization of the higher-order genomic structure—the G-quadruplex (G4)—in the G/C-rich proximal region of the MYC promoter is an established approach to decreasing transcription and facilitating tumor lysis [32,33]. Negative superhelicity induced by MYC transcription due to translocation or overexpression, as found in NHL, can promote local unwinding of the G/C-rich promoter region, which allows for the formation of G4s. G4s are made up of two or more stacked tetrads, formed by the Hoogsteen hydrogen bonding of four guanines, and are stabilized by monovalent cations, such as K^+^. Putative G4-forming regions have at least four runs of two or more (most often three or more) consecutive guanines (G-tracts) separated by varying nucleotides that comprise the loop structures. The structures are classified by their loop directionality, length, and constitution [32,33,34]. The MYC promoter contains six contiguous runs of three or more continuous guanines. While several G4 isoforms have been described within the MYC promoter G-rich region, the physiologically relevant isoform has been identified to form from the first four contiguous runs, termed G4_1-4_ [30,32,34,35,36].

**Figure 1:**
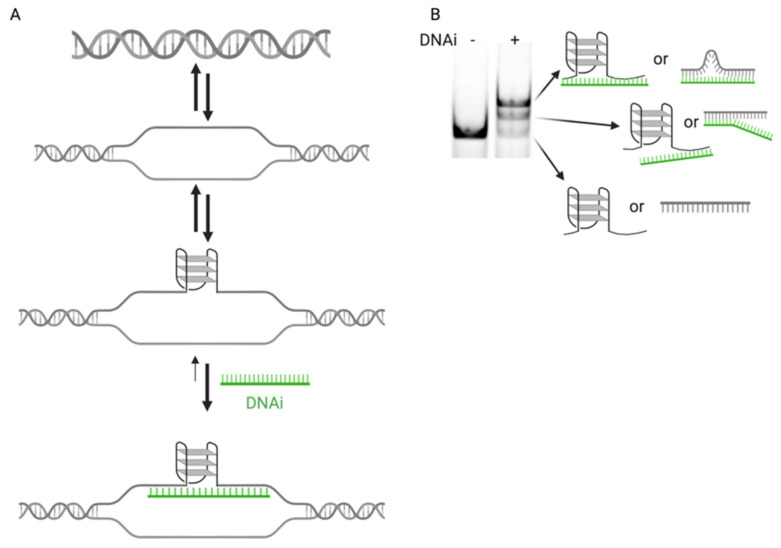
Proposed DNAi mechanism of action for MYC G4 recognition. (**A**) dsDNA opens up on a transient basis due to superhelical stress and is capable of forming a higher-order G4 structure. Adding DNAi that complements the regions flanking the G4-forming promoter section can shift the equilibrium to maintain more G4 formation, enhancing the silencing function within the MYC promoter. (**B**) EMSA separation of the higher-order DNA species can differentiate between unbound linear or G4 DNA, DNA wherein the DNAi complements the G4-forming region on one flank, and DNA where the DNAi captures both flanking regions.

While many molecules have been developed that target G4s [37], very few have demonstrated selectivity for an individual promoter G4 [38]. Only a select few have advanced to clinical trials [34,39], including one pan-G4-stabilizer in current trials for BRCA-mutated breast cancers [40]. However, no preclinical or clinical agent has yet been described with a high degree of selectivity. As a means to overcome specificity constraints, our group pioneered a clamped DNA interference (DNAi) approach to take advantage of the “druggability” of the DNA structure in the MYC promoter [35]. Our previously described clamp complements the 5′ and 3′ regions of the promoter that flank the G4 and holds them at a distance of 18 Å with an abasic linker. The oligonucleotide clamp was shown to be specific for the physiologically relevant structure forming from the first four of six contiguous runs of continuous guanines (G4_1-4_), to induce its formation, and to dose-dependently downregulate MYC promoter activity by up to 71%, in vitro. While the originally described clamp utilized an abasic linker to span the 18 Å, in order to enable more flexible modification of the oligonucleotide, as well as to increase its scalability in a cost-effective manner from several commercial sources, we sought to replace the linker with thymines. Thymines were chosen due to their lack of involvement in G::C basepairing. Five thymines maintain the 18 Å of the original clamp. Plus or minus two thymines (3T-7T), for a distance of ±7.2 Å, within the linking region were explored in the development of the clamp into a DNAi (Figure 1A); 1T was included as a negative control, as a linker of 3.6 Å does not fit the model of enabling G4-formation.

## 2. Result

### 2.1. Biophysical Characterization of DNAi Binding to the MYC G4

**Figure 2:**
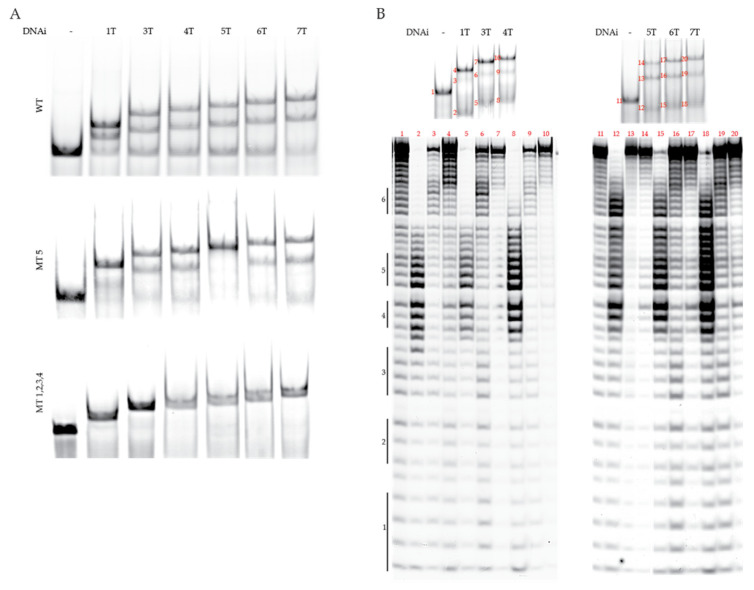
DNAi optimization for MYC G4 recognition. (**A**) Electromobility shift assays (EMSAs) were performed with the DNAi array with WT Pu46 DNA (top), G4_1-4_-forming MT 5 DNA (middle), and non-G4 forming MT 1,2,3,4 DNA (bottom). As determined in the development of clamp a [35], the supershifted (middle band) DNA represents the DNAi complementing the 5′ G4-flanking region, while the suprashifted (upper band) DNA represents a DNAi:MYC G4. (**B**) DMS footprinting clarifies the G:::G bonding of Pu46 with each DNAi, as isolated from each EMSA band. The six contiguous runs of continuous guanines within the Pu46 sequence are denoted to the left of the footprints. Linear DNA is noted in Bands 1–6, 8, 9, 11, 12, 15, 16, 18, and 19. G4_1-4_ formation is evident in Bands 7, 10, and 14, indicated that DNAi 3T, 4T, and 5T can stabilize the physiologically relevant structure. A protection pattern ascribed to a mixture of G4 isoforms is noted in Bands 13, 17, and 20.

The replacement of an abasic linker with thymines affords a cost-effective means to increase flexibility in the linker distance between the two flanking complementary regions of the DNAi, as commercial costs were cut more than 20-fold. Therefore, we examined the binding and G4-stabilization of an array of DNAi with linkers ranging from 3.6 to 25.2 Å with 1 or 3–7 thymines (named 1T, 3T–7T). The DNAi array was incubated with the MYC G-rich, G4-forming Pu46 sequence, which was then annealed to form a G4 and subjected to EMSA (Figure 1B and Figure 2A, top). All of the DNAi demonstrated both super- and suprashifted bands, indicating both complementarities to the G4-flanking region to form dsDNA, and either recognition of a higher-order G4 structure or capture of both the 5′- and 3′-flanking sequences, respectively (Figure 1B). G4 and dsDNA formation was confirmed by electronic circular dichroism from the mixture of structures, wherein a positive Cotton effect at 262 nM coincides with a parallel G4 and the presence of a shoulder Cotton effect from 270 to 280 nM in the presence of all DNAi confirms dsDNA binding (Figure S1). Super- and suprashifted banding was noted with the MYC MT5 sequence, which isolates the physiologically relevant G4_1-4_ structure (Figure 2A, middle), while only double-stranded binding was noted with the G4 knockout MYC MT1,2,3,4 sequence (Figure 2A, bottom). This band pattern and sequence specificity were also noted with the original clamp a [35]. Notably, 1T and 5T demonstrated the greatest induction of the suprashifted band when incubated and annealed with both the WT and MT 5 sequences.

Through previous experience, it was noted that the supershifted bands noted on EMSA represented dsDNA formed through complementing the 3′ flanking region of the G4-forming Pu46 sequence and the suprashifted band represented a G4 captured by dsDNA formation on both flanks. It is, however, plausible that the suprashifted band can occur without G4 formation, such as by stabilizing a loop flanked by two dsDNA complementing regions (Figure 1B). Thus, in order to assess the nature of the DNA structure separated and represented by each band, Pu46 was incubated with each DNAi at a 1:1 ratio, the species were separated by EMSA, isolated from the gel, and subjected to DMS footprinting (Figure 2B). G4_1-4_ formation is noted in the suprashifted bands when Pu46 was incubated with 3T (Lane 7, full protection noted in the first three runs, and partial protection in Runs 4 and 5), 4T (Lane 10, protection notable in all six runs of contiguous guanines), and 5T (Lane 14, protection noted in the latter three guanines of the first guanine run, the second and the third; partial protection noted in Run 4 and hypercleavage evident in Run 5). A less clear cleavage pattern, and thus G:::G bonding pattern to form a G4, was clarified with Pu46 + 1T (Lane 7, no pattern of protection noted), 6T (Lane 17, minimal protection in Runs 1 and 3), or 7T (Lane 20, minimal protection in Runs 1 and 3) incubation. In particular, the suprashifted EMSA band formed in the presence of 5T is most in agreement with G4_1-4_ formation and in the presence of 1T is consistent with an ssDNA loop, as seen by DMS tagging and subsequent cleavage at all guanines in the sequence, rather than a higher-order G4.

### 2.2. DNAi Therapeutic Potential

**Figure 3:**
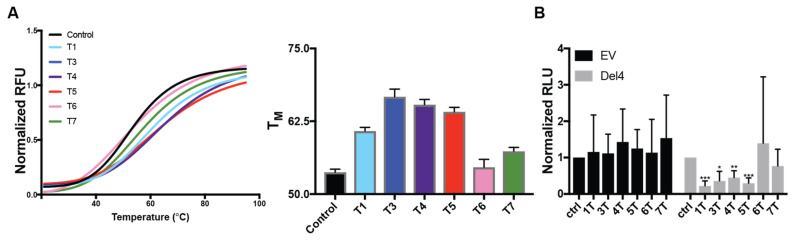
Therapeutic potential of DNAi. Thermal stabilization of MYC G4 formation by the DNAi, as measured by the FRET Melt^2^ assay (**A**). Melting curves (left) were analyzed by nonlinear regression methods to determine the T_M_ (right). Equimolar ratios of DNAi 3T, 4T, and 5T demonstrated >10 C increases in thermal stability of MYC G4-forming Pu46, 1T increased thermal stability by 7 °C, while 6T and 7T’s effects were negligible (<4 °C). Experiments were performed in triplicate, each with technical duplicates. (**B**) HEK-293 cells were transiently transfected with a promoterless (EV) or MYC promoter-containing (Del4) luciferase plasmid in the absence and presence of each DNAi at a 1:1 ratio. The effects of the DNAi on promoter activity were examined 48 h later. No DNAi affected the promoterless vector; DNAi 1, 3, 4, and 5T all significantly decreased MYC promoter activity. * *p* < 0.05, ** *p* < 0.01, *** *p* < 0.001; experiments were performed in triplicate.

Each of the DNAi was further incubated with dual-labeled Pu46, which was subjected to the FRET Melt^2^ assay (Figure 3A) [41]. The T_M_ of Pu46 incubated with vehicle control was 53.8 ± 0.5 °C, and the ΔT_M_ after incubation with the DNAi ranged from 0.9 to 13 °C. 3T, 4T, and 5T demonstrated the most marked (ΔT_M_ > 10 °C) increases in T_M_ to 66.7 ± 1.3, 65.4 ± 0.9, and 64.2 ± 0.8 °C, respectively. 1T increased the T_M_ to 60.8 ± 1.8 (ΔT_M_ = 7 °C), whereas the changes in T_M_ with 6T and 7T were negligible (ΔT_M_ < v4 °C to 54.6 ± 1.7 and 57.4 ± 1.8, respectively). Subsequently, HEK-293 cells were transiently transfected with either promoterless luciferase vector (EV) or one containing the MYC promoter (Del4) in concert with equimolar DNAi, as previously reported for clamp a [35] (Figure 3B). DNAi 1T, 3T, 4T, and 5T all significantly decreased promoter activity in the Del4 plasmid, in agreement with the FRET Melt^2^ findings and further corroborating the enhanced predictive nature of the modified FRET assay. 1T and 5T most markedly decreased promoter activity by 81 and 76%, respectively. Taken together, 1T appears to complement the 5′ and 3′ flanking regions of the MYC promoter G4, facilitating a single-stranded intervening loop, while 3–5T appear to complement the flanks and enable G4 formation. From a biological perspective, as measured by the luciferase assay, alteration of a noncanonical dsDNA structure of this region to either a G4 or ssDNA loop may modulate MYC expression. Follow-up studies were performed on these four sequences in a more biologically intact system.

### 2.3. DNAi Cellular Activity

**Figure 4:**
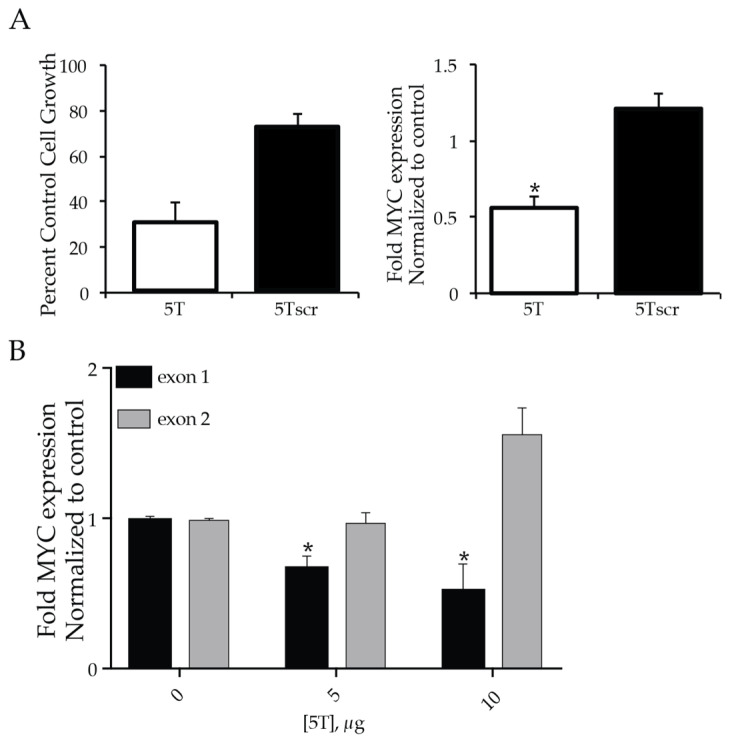
Efficacy of DNAi 5T in Burkitt’s lymphoma cell lines. (**A**) DNAi 5T or 5Tscr (500 ng) was incubated with RAJI cells for 24 h and the effects on cell viability (left) or MYC regulation (right) were measured. 5T, but not 5Tscr, decreased both RAJI cell viability and MYC mRNA expression. In the CA46 BL cell line, lacking G4-control on the majority of MYC expression, no effects were noted on cell viability with up to 10 μg of DNAi. However, (**B**) the CA46 exon test demonstrated a significant dose-dependent decrease in MYC expression in mRNA containing exon 1, which is still under the control of the MYC promoter G4, but not from exon 2 lacking G4-control. Experiments were performed in duplicate with technical triplicates. * *p* < 0.05 as compared to untreated control.

Burkitt’s lymphoma (BL) is driven by a translocation that puts MYC expression under the regulation of an immunoglobin gene [20]. In the RAJI BL cell line, the translocation between chromosomes 8 and 14 results in increased transcription of the MYC gene and maintains regulation by the MYC promoter G4. In the CA46 BL cell line, the chromosome 8:14 translocation similarly results in increased MYC transcription, but there is a loss of G4-mediated transcriptional control for most MYC expression. These paired cell lines are often used to discriminate between MYC G4-related therapeutic effects. DNAi 1T, 3T, 4T, and 5T, having demonstrated downregulation of MYC promoter activity, were examined further for MYC G4-stabilization and intracellular activity in RAJI and CA46 lymphoma cell lines. Their cytotoxic effects were examined for 72 h, and their effects on MYC transcription were monitored (Figure 4 and Figure S2). The cellular viability of RAJI and CA46 cells were dose-dependently modulated by 1T, 3T, and 4T in a manner that does not discriminate between the predominant MYC G4-maintaining RAJI cells, and the MYC G4-lost CA46 cells (Figure S2). While RAJI cells demonstrated decreased viability, as compared to CA46 cells at 30 and 100 ng of DNAi 1T, 3T, and 4T, at higher doses, both cell lines were similarly affected. Examination of changes in MYC transcription 500 ng DNAi, the ~IC50 at 72 h, did not reveal any remarkable changes in either cell line.

In contrast, 5T mediated a dose-dependent decrease in RAJI cell viability with no significant effects in the CA46 cell line. The 72 h IC_50_ of 5T in RAJI cells is 358 ± 2.6 ng, whereas CA46 were unaffected at concentrations up to 10 μg. To fully explore the cellular effects of 5T, described below, we designed a scramble control (5Tscr) of the same length and containing the same nucleotides. 5Tscr was subjected to EMSA and FRET Melt^2^ analysis. No super- or suprashifting was noted by EMSA (Figure S3); the ΔT_M_ determined by FRET Melt^2^ with 5T scr incubated with dual-labeled MYC G4 DNA was 0.9 °C. From these consistent data, 5T scr was further used as a negative DNAi control. 5Tscr does not modulate the viability of RAJI cells at doses up to 1 μg. The effects of 500 ng of 5T and 5Tscr in RAJI cells were compared for both viability and effects on MYC transcription (Figure 4A). DNAi 5T significantly (* *p* < 0.05) decreased both RAJI viability by 75% and MYC expression by 48%, whereas 5Tscr did not mediate any significant change.

No changes were noted in CA46 viability with the 5T or 5Tscr DNAi up to 10 μg, in agreement with the proposed mechanism of DNAi action. A benefit of studying the CA46 cell 5 is the ability to run an ‘isogenic’ qPCR assay termed the CA46 exon test, which can differentiate changes in MYC expression within the CA46 cell line under the control of the endogenous promoter G4 (exon 1 only) as compared to the translocated promoter lacking a G4 (exon 2) [38,39,42]. Using this assay, we examined the effects of G4-mediated changes in MYC expression as demonstrated by the dose-dependent decreased expression in CA46 cells of MYC mRNA containing exon 1, but not exon 2, by DNAi 5T (Figure 4B), no changes in either exon were noted with 5Tscr.

### 2.4. DNAi 5T Selectivity and Specificity for the MYC G4

**Figure 5:**
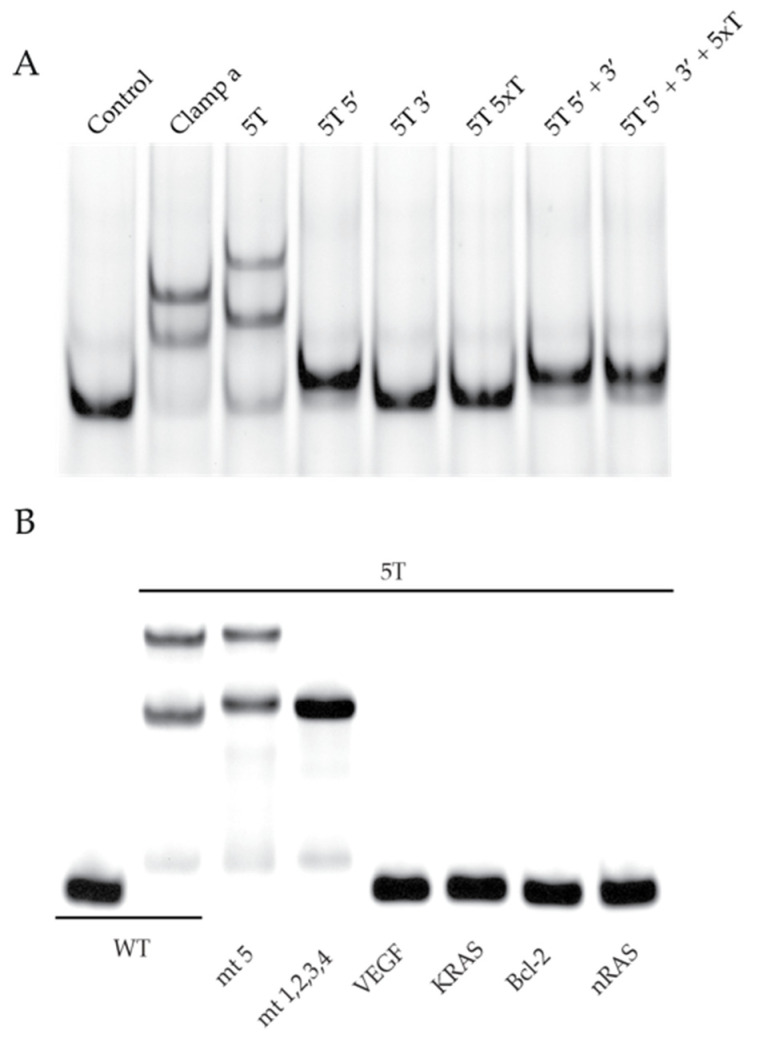
DNAi 5T selectivity and specificity for MYC G4_1-4_. (**A**) 5T binding to WT Pu46 was compared to clamp a and to its partitioned components. 5T shifting was similar to that of clamp a, albeit heavier due to the five thymes, as compared to the abasic linker. Notable recognition of Pu46 was not evident with any component of 5T. (**B**) FAM-labeled 5T was used to examine binding to an array of G4-forming sequences, including those from the MYC (WT and MT5, as well as the non-G4-forming MT1,2,3,4), VEGF, KRAS, Bcl-2, and nRAS promoters were examined.

DNAi 5T encompasses approximately the same distance of 18 Å as the originally published clamp a [35]. The binding of 5T to the WT MYC G4-forming DNA was compared to that of clamp a (Figure 5A) by EMSA. Both the super- and suprashifted banding patterns were comparable, although those associated with 5T showed a slower migration pattern due to the weight and size of the five thymines, as compared to the abasic linker. Moreover, it was previously demonstrated that the 3′, 5′, and linking regions were all required to be both present and physically connected for clamp a recognition of the MYC G4_1-4_ [35]. DNAi 5T was dissected to its three components (5T 5′, 5T 3′, and 5T 5xT) to examine if the same requirement was maintained (Figure 5A). Minorly shifted bands are visible when the 5′ 5T sequence, complementing the 3′ flank of the MYC G4 is present, but super- and suprashifting EMSA patterns that matched clamp a were noted only when all three components were present and physically connected. The selectivity of 5T for the MYC G4 isoform was shown again by comparative EMSAs with the WT, MT5, and MT1,2,3,4 sequences of the MYC promoter, and specificity for the MYC G4 was demonstrated by incubating DNAi 5T at equimolar concentrations with the G4-forming sequences for the VEGF, KRAS, BCl-2, and NRAS promoters. Unsurprisingly, given the lack of complementarity between the DNAi and the other promoter G4 sequences, no shifted bands were noted (Figure 5B).

### 2.5. DNAi 5T Recognition of the MYC G4

**Figure 6:**
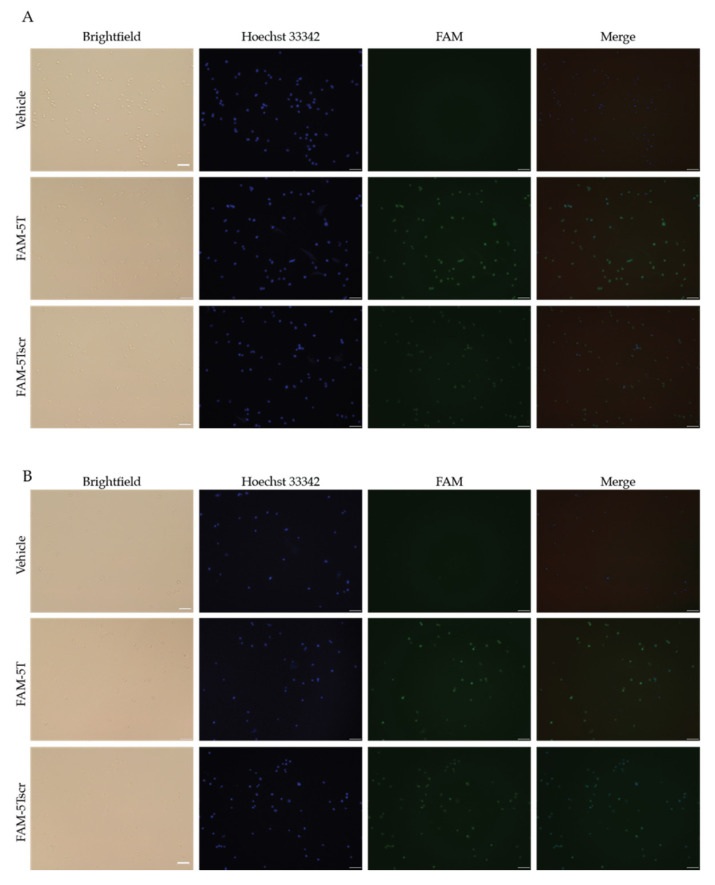
DNAi 5T nuclear localization is enhanced with MYC G4 stabilization by NSC 338258 in RAJI Burkitt’s lymphoma cells. RAJI cells were incubated with the indicated DNAs alone (**A**) or in the presence of 2.5 μM NSC 338258 (**B**) for 24 h before fluorescence visualization. No FAM accumulation is noted in the transfection vehicle controls without DNAi. 5T is noted to accumulate in the cells and in the nuclei ~80% more than 5T scr in the absence of NSC 338258. Co-incubation with the MYC G4-stabilizing compound increases the mean FAM penetration ~2-fold for 5T, with no measurable change in 5Tscr accumulation. Images are representative of duplicate experiments with triplicate images collected within each experiment.

RAJI cells were incubated with FAM-labeled DNAi 5T or 5Tscr for a day before fixation, permeabilization, and visualization for DNAi nuclear penetration, as measured by confocal microscopy, as compared to a transfection vehicle control (Figure 6A). FAM-labeled 5T had comparable binding affinity to the MYC G4 as unlabeled 5T (Figure S4). Both 5T and 5Tscr demonstrated nuclear uptake, as evidenced in the overlay wherein FAM localization coincided with Hoescht 33342 stained nuclei. ImageJ analysis of FAM and Hoescht means within each confocal image illustrate 1.8-fold higher mean intranuclear concentration of 5T, as compared with 5Tscr, within the RAJI cells (Table 1).

NSC 338258 is a compound that was previously demonstrated to decrease MYC transcription by stabilization of the MYC promoter G4 in the RAJI cell line [42]. RAJI cells were incubated for a day without (Figure 6A) or with (Figure 6B) the 2.5 μM NSC 338258 (the 24 h IC_50_) alone or with vehicle, 5T or 5Tscr before fixation, permeabilization, and visualization. Enhanced nuclear localization was noted for DNAi 5T, but not 5Tscr. Quantification of the enhanced mean FAM penetration, as compared to Hoescht intensity, demonstrated an overall 2-fold increase for 5T, but no change for 5Tscr, in agreement with the mechanism of action for NSC 338258-mediated regulation of the MYC G4 and subsequent transcriptional control.

## 3. Discussion

In the current study, we converted our previously described G4-stabilizing clamp [35], an oligonucleotide complementing the 5′ and 3′ flanking regions of the MYC promoter G4 connected with an abasic polyethylene glycol phosphate linker, to one that utilized one to seven thymines as a linker. Using biochemical analyses, we demonstrated that the pure oligonucleotide interfering DNAs (DNAis) with three, four, or five thymines were able to induce and stabilize the target G4 structure and regulate the MYC promoter in a luciferase assay. Interestingly, DNAis with one, six, or seven thymines seemed to stabilize open loops in the G4-forming region, but 1T stabilized the loop and lowered MYC promoter activity. Only DNAi 5T, however, modulated lymphoma toxicity with correlating changes in MYC transcription in the RAJI cell line and passed the CA46 exon test. Additionally, DNAi 5T was further shown to accumulate in the nuclei of lymphoma cells, additively with the MYC G4 stabilizing compound NSC 338258 [42], and it remains the most effective DNAi to target the MYC G4-forming promoter region. The phenomenon wherein 1T stabilized an open loop opens the field for a novel DNA target but is beyond the purview of the current report. Further studies examining the dose-dependent combination of NSC 338258 and DNAi 5T would likely provide interesting insight into the mechanism of action and potential therapeutic benefit, but unfortunately, NSC 338258 is no longer available from the NCI DTP program.

The MYC promoter G-rich region contains six contiguous runs of three or more continuous guanines. Although a number of equilibrating higher-order G4s have been described under extracellular conditions, G4_1-4_ has been shown to form under nuclearly relevant supercoiled positions and thus to be the best candidate structure to be formed in cells [32,34,41,42,43]. The development of therapeutics targeting the MYC promoter G-rich region have not consistently focused on G4_1-4_, either through screens biased towards other structures—most commonly G4_2-5_ [44,45]—or through techniques that did not facilitate predominantly G4_1-4_ formation [46,47], leading to a high failure rate for therapeutic development. Our previous development of clamp a confirmed the G4 formed from the first four contiguous runs to be the silencing structure within the G-rich region [35] and thus to be the primary target to best facilitate MYC downregulation. G4_1-4_ remained the molecular target for DNAi development in the current study, as confirmed using both mutated DNA (MT5) and the FRET Melt^2^ method we used to examine thermal stability of the MYC promoter G4s, which was recently optimized to be a better predictor of in cell G4 stabilization and subsequent MYC downregulation as confirmed by the CA46 test [38,41,48]. The optimized DNAi 5T, indeed, mediated inhibition of lymphoma cell viability in correlation with MYC downregulation through apparent promoter G4 stabilization.

Research into G4s as molecular targets, and the development of small molecules, has been undertaken for almost 20 years. While small molecules selective for particular G4s have been identified [37,41,49], they still demonstrate a measure of promiscuity for similar structures. DNAis have been developed for a non-G4-related region of the Bcl-2 promoter, where they have advanced to clinical trials [48,49]. The DNAi described herein, which clamps the promoter region flanking the physiologically relevant MYC promoter G4, overcomes the limitations of small molecules [35]. Further efforts to develop the DNAi as a first-in-class therapeutic targeting the MYC promoter G4 are ongoing, with a focus on enhancing cellular and nuclear accumulation, intracellular stability, and overall combination regimens. Although our current DNAi efforts are aimed at the MYC promoter, as a high-value molecular target for anticancer therapeutics, the approach and technology can be applied to an array of noncanonical DNA structures with fully characterized, physiologically relevant, structures.

MYC has both transcription and nontranscription related functions and is upregulated in a wide array of oncogenic lesions [50,51]. It has been shown to be a high-value molecular target with a wide potential therapeutic index that has proven difficult to selectively and directly target [30,31,52,53,54]. Our DNAi approach enables specific and direct downregulation of MYC expression, with potential efficacy in MYC-reliant cancers, such as non-Hodgkins lymphoma (NHL). MYC is aberrantly regulated or expressed in aggressive NHLs, such as poor prognosis DLBCL and MCL, as well as in the majority of BL—the model system used in the current study. Decreasing MYC expression has been shown to significantly impact the viability of NHL tumors. Efforts to develop the MYC G4-targeting DNAi 5T will continue in NHL but are more broadly applicable to other MYC-reliant cancers, such as breast [55,56,57], lung [58], colon [59], and many more cancers [60].

## 4. Materials and Methods

Materials. Oligonucleotides were synthesized and purchased from Eurofins MWG Operon, LLC (Louisville, KY, USA) (Table 2. Acrylamide/bisacrylamide solution (29:1) and ammonium persulfate were purchased from Bio-Rad Laboratories (Hercules, CA, USA); *N*,*N*,*N*′,*N*′-tetramethylethylenediamine was purchased through Fisher Scientific (Pittsburgh, PA, USA). All other chemicals were purchased from Sigma-Aldrich (St. Louis, MO, USA).

Electromobility Shift Assay (EMSA). G4-forming DNA (naked or fluorescently labeled), sequence as indicated, was diluted to 1 μM in 25 mM KCl plus 50 mM Tris–HCl (pH 7.4) in the absence or presence of equimolar DNAi (naked or fluorescently labeled) and incubated at room temperature for 30 min. G4 annealing was performed by heating to 95 °C for 5 min and rapidly cooling for 5 min. Upon addition of nondenaturing loading dye, the samples were loaded onto a 10% native polyacrylamide gel. After running at 100 V, the gels were visualized with blue light LED using a Typhoon 5 BioMolecular Imager (GE Health Sciences, Pittsburgh, PA, USA). The image was aligned horizontally based on the location of the wells.

FRET Melt^2^. 5′-FAM and 3′-TAMRA labeled Pu46 was diluted to 2 μM in 10 mM sodium cacodylate (pH 7.4) plus 90 mM LiCl, 10 mM KCl, and 10% glycerol, and annealed to form a G4 as described above. G4 DNA was added to a 96-well PCR plate with or without DNAi (2 μM). Fluorescence was recorded from 20 to 95 °C, at every degree after a 10 s hold on a Bio-Rad CFX96 real-time thermocycler (Hercules, CA, USA). The fluorescence values were normalized to each well’s 20 °C fluorescence measured, and nonlinear transformation was performed using GraphPad Prism (San Diego, CA, USA) to determine the G4 T_M_.

Dimethyl sulfate (DMS) footprinting. Cy5-labeled MYC G4-forming DNA (Pu46; 10 μM), either with or without equimolar concentrations of clamp (10 μM), was added to a buffer of 25 mM KCl and 50 mM of Tris–HCl. Dimethylsulfate (DMS, 10%) in 40% ethanol was added to each sample to a final concentration of 0.5% DMS and 2% ethanol, which was incubated at room temperature for 15 min before adding calf-thymus DNA (1 μg) and gel loading buffer (bromophenol blue, 0.005% final concentration). Samples were then electrophoresed as described above with EMSA, and the isolated bands were excised and extracted with gel elution buffer (0.4 M ammonium acetate, 1 mM MgCl_2_, 0.2% SDS) in a 37 °C water bath overnight. The supernatant was collected, and DNA was precipitated in 75% ethanol with 3 M sodium acetate at −20 °C overnight. Samples were centrifuged at 15,000 rpm for 30 min, and the supernatant was discarded. DNA pellets were air-dried for 30 min before they were resuspended in 10% piperidine to cleave DMS tagged DNA, heated at 95 °C for 30 min, and snap-cooled on ice. DNA was precipitated in ethanol/sodium acetate as above, then dried and washed twice with water. Finally, DNA was suspended in 15 μL of DMS dye (80% formamide, 10 mM NaOH, 0.005% bromophenol blue), heated to 95 °C for 5 min, cooled on ice immediately, and run on a 16% denaturing gel with 7 M urea at 2 W, overnight at 4 °C. The gel was visualized as described above.

Cell lines and culturing methods. Nontransformed HEK-293 and RAJI and CA46 Burkitt’s lymphoma cell lines were purchased from ATCC (Manassas, VA, USA) and were maintained in exponential growth in either Dulbecco’s minimal essential media (DMEM, HEK-293) or RPMI-1640 (RAJI and CA46) media supplemented with 10% fetal bovine serum (Sigma-Aldrich, St. Louis, MO, USA) and 1× penicillin/streptomycin in suspension culture held at 37 °C and 5% CO_2_ until experimental use.

Luciferase Assay. HEK-293 cells in exponential growth were seeded in 24-well plates at 8 × 10^4^ cells per well and allowed to attach overnight in a 37 °C humid incubator supplied with 5% CO_2_. The following day, the cells were cotransfected with 250 ng of the promoterless empty pGL4.17 vector plasmid (Promega, Madison, WI, USA) or the MYC promoter containing Del4 plasmid (gift from Burt Vogelstein, Plasmid No. 16604, as supplied by AddGene, Cambridge, MA, USA) plus renilla plasmid (pRL-SV40, 100 ng, Promega), either without or with DNAi (500 ng), using Fugene HD (Promega) in a 3:1 ratio. Forty-eight hours post-transfection, cells were lysed in passive lysis buffer (Promega), placed in a −20 °C freezer, thawed, and put through one more freeze/thaw cycle to promote cell lysis. Luciferase was measured with the Dual-Luciferase Assay Kit (Promega) on a Lumat LB9507 luminometer. Experiments were completed in biological triplicate. Within each biological replicate, the firefly luciferase was normalized to its control renilla RLU, and the DNAi effects were normalized to no DNAi control. The effect of each DNAi was compared to control using a two-tailed Student’s *t*-test to determine statistical significance.

Confocal microscopy. RAJI cells were seeded in non-tissue culture treated chamber slides at a concentration of 250,000 cells/mL overnight before the addition of 500 ng DNAi 5T or 5T scramble formed into micelles with JetPrime transfection reagent (Polyplus, NY, USA) alone or with 2.5 μM NSC 338258. After 24 h of incubation, cells were incubated for 10 min with Hoescht 33342 directly in the media before fixation with 3.7% paraformaldehyde and visualization with a Leica SP8 confocal microscope at 20× magnification. Image J (NIH, Bethesda, MD, USA) was used to quantify the mean intensity for each fluorescent signal in the DAPI and FITC (e.g., Hoescht and FAM) channels. The experiment was repeated in duplicate with triplicate images obtained within each experiment.

Cell viability and qPCR. To measure cellular viability, RAJI or CA46 cells were seeded in non-tissue culture treated 96-well plates at a cell density of 200,000 cells per well in 80 μL and incubated at 37 °C overnight. A 20 μL volume of micelles formed with the described (DNAi) and JetPrime transfection reagent at a 2:1 ratio were added to the cells and allowed to incubate for 24 or 72 h, as indicated, before 20 μL of MTS + 5% PMS were added to the wells and allowed to incubate for 2–4 h, as described previously [38]. RAJI and CA46 cells were seeded at a density of 250,000 cells per mL in a non-tissue culture treated plate overnight before incubation with the DNAi, as described. At 24 or 72 h, as indicated, cells were collected, washed with PBS and the RNA was isolated with the GeneJET RNA purification kit (Thermo Fisher, Waltham, MA, USA). Up to 2 μg of RNA was reversed transcribed using the qScript RT kit from Quantabio (Beverly, MA, USA) before quantitative real-time PCR was performed on a Bio-Rad CFX96 thermocycler (Hercules, CA, USA) using FAM-labeled TaqMan primers from Applied Biosystems (Carslbad, CA, USA), as previously described for MYC and GAPDH [38]. The ΔΔCt method was used to calculate changes in expression mediated by the DNAi, as normalized to vehicle-treated controls. Due to the markedly lower expression, 10-fold more input cDNA is used to monitor exon 1-containing expression in CA46 cells, as compared to exon 2-containing mRNA. Statistical analysis was performed with GraphPad Prism software using either a Student’s t-test for the single-dose experiments or a one-way ANOVA with a post-hoc Tukey’s test for the dose-dependent CA46 exon test.

## Figures and Tables

**Table 1:** Intranuclear accumulation of DNAi in RAJI cells.

DNAi	NSC338258	Mean Hoescht Intensity	Mean FAM Intensity	Hoescht_mean_/FAM_mean_
5T	-	4.1	6.4	1.6
5Tscr	-	3.9	5.6	0.9
5T	+	2.3	7.0	3.1
5Tscr	+	4.4	6.1	0.9

**Table 2:** DNA sequences used in the current study. Continuous runs of three or more guanines are underlined.

Oligonucleotide	5′-3′ Sequence
Pu46/WT	GCGCTTATGGGGAGGGTGGGGAGGGTGGGGAAGGTGGGGAGGAGAC
MT5	GCGCTTATGGGGAGGGTGGGGAGGGTGTGGAAGGTGGGGAGGAGAC
MT1,2,3,4	GCGCTTATGGTGAGTGTGGTGAGTGTGGGGAAGGTGGGGAGGAGAC
VEGF	CCGGGGCGGGCCGGGGGCGGGGTCCCGGCGGGGCG
KRAS	GCGGGGAGAAGGAGGGGGCCGGGCCGGGCCGGCGGGGGAGGAGCGGGGGCCGGGCC
Bcl-2	AGGGGCGGGCGCGGGAGGAAGGGGGCGGGAGCGGGGC
NRAS	GTGGGAGGGGCGGGTCTGGGTGCGGCC
Clamp a	CTCCTCCCCACCTTCCCC[C-18]ATAAG
1T	CTCCTCCCCACCTTCCCCTATAAG
3T	CTCCTCCCCACCTTCCCCTTTATAAG
4T	CTCCTCCCCACCTTCCCCTTTTATAAG
5T	CTCCTCCCCACCTTCCCCTTTTTATAAG
5Tscr	GACCTTCCATCTCCCACTCCTTCCTATTA
6T	CTCCTCCCCACCTTCCCCTTTTTTATAAG
7T	CTCCTCCCCACCTTCCCCTTTTTTTATAAG
5T 5′	CTCCTCCCCACCTTCCCC
5T 3′	ATAAG
5T 5xT	TTTTT

## Data Availability

Not applicable.

## Supplementary Materials

The following are available online, Figure S1: ECD evaluation of DNA topology, Figure S2: Activity of DNAi 1T, 3T and 4T in lymphoma cells, Figure S3: Evaluation of 5Tscr G4 recognition, Figure S4: Affinity of unlabeled and labeled 5T.

## Sample Availability

Samples of the compounds are not available from the authors.
