# Supplementary material for: Targeted Downregulation of MYC through G-quadruplex Stabilization by DNAi
Source: Molecules. 2021 Sep 13;26(18):5542. doi: 10.3390/molecules26185542 (PMC8464964; doi:10.3390/molecules26185542)
Supplement: Supplementary file 1 [file molecules-26-05542-s001.zip › molecules-1284315-supplementary.pdf]

Supplementary Materials

Targeted Downregulation of MYC Through G-quadruplex Stabilization by DNAi

Alexandra Maria Psaras [1], Katarina T. Chang [1], Taisen Hao[2] and Tracy A. Brooks [1,2,*]

[1] Department of Pharmaceutical Sciences, School of Pharmacy and Pharmaceutical Sciences, Binghamton University, Binghamton, NY 13902, USA

[2] Department of BioMolecular Sciences, School of Pharmacy, University of Mississippi, University, MS, 38677, USA

* Department of BioMolecular Sciences, School of Pharmacy, University of Mississippi, University, MS, 38677, USA

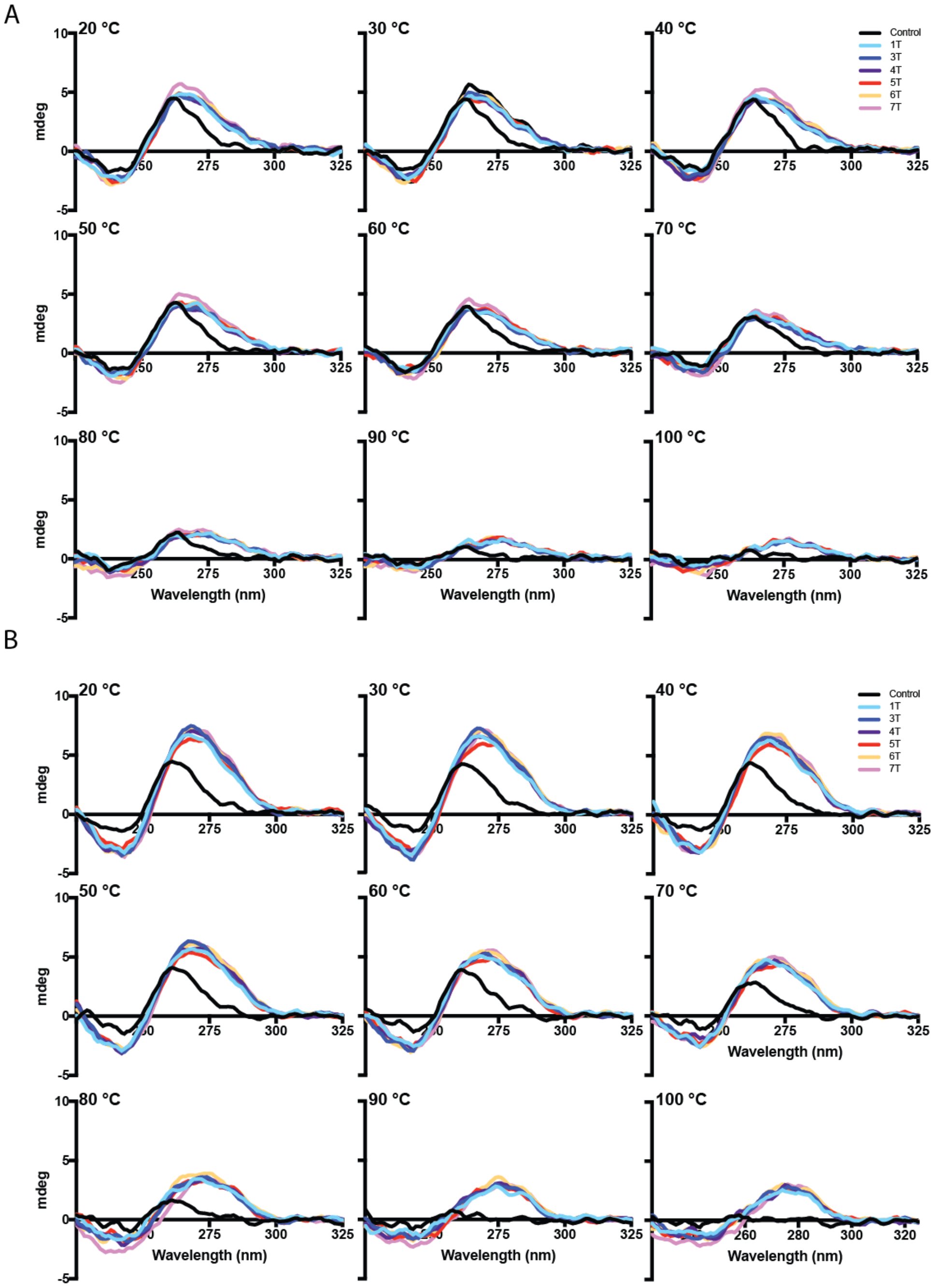

Figure S1. ECD evaluation of DNA topology. The effect of DNAi binding to Pu46 at (A) 1 and (B) 2 equivalents was examined by ECD from 20-100 °C in the presence of 25 mM KCl and 10 mM Tris HCl buffer. Control Pu46 formed a stable G4 alone and in the presence of each DNAi, as evidenced by a positive Cotton effect at 262 nM. DNAi also formed dsDNA with the flanking regions of the DNAi, as noted with the shoulder Cotton effect from 270-280 nM. More G4 formation was noted in the presence of 2 equivalents of DNAi, marked by an increased mdeg.

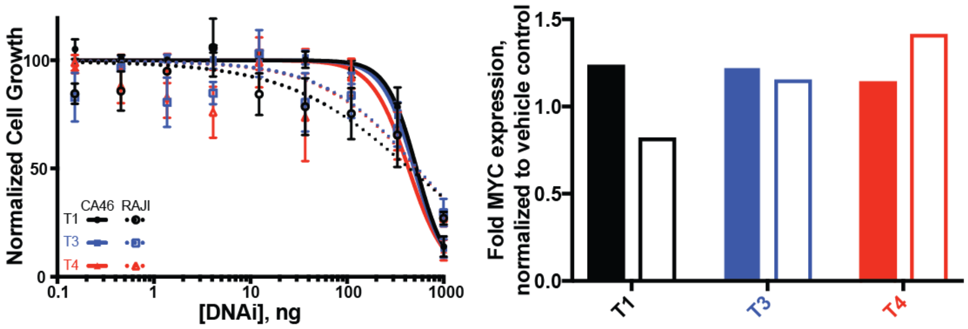

Figure S2. Activity of DNAi 1T, 3T and 4T in lymphoma cells. (A) 72 hour evaluation of DNAi cytotoxic activity in RAJI (open symbol, dashed line) and CA46 (filled symbol, solid line) cell lines highlighted no preference for RAJI cells. (B) Examination of transcriptional modulation of MYC in CA46 (filled) and RAJI (open) cells did not demonstrate any marked decreases. Experiments were performed in at least duplicate with internal duplicates.

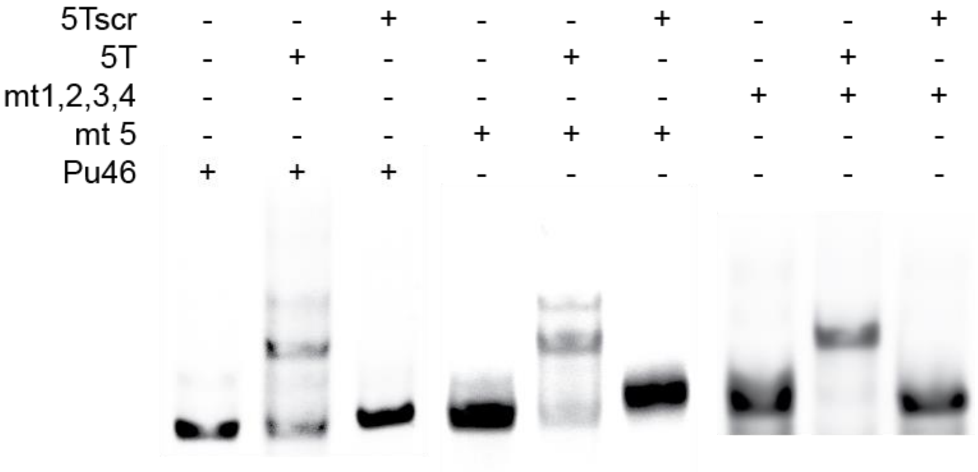

Figure S3. Evaluation of 5Tscr G4 recognition. Control DNAi 5Tscr was examined for binding to the MYC G4 (pu46), $G4_{1-4}$ (mt 5) or G4-forming region (mt1,2,3,4) by EMSA. No binding was noted to any of the sequences. DNAi 5T was included as a positive control.

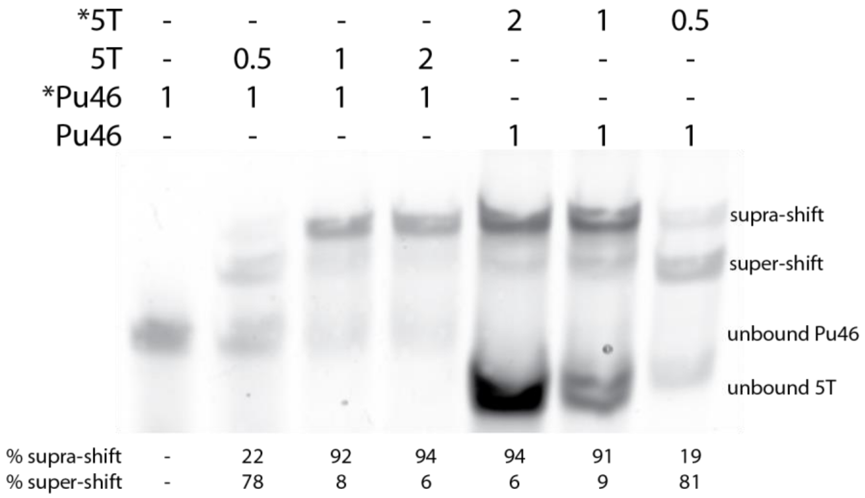

Figure S4. Affinity of unlabeled and labeled 5T. FAM labeled Pu46 was incubated with unlabeled DNAi 5T, or vice versa, at ratios of DNAi:G4DNA of 0.5, 1, or 2 to compare G4 affinity. Comparing the distribution of super- and supra-shifted bands (% at bottom), labeled or unlabeled 5T had the same affinity for Pu46. Unbound Pu46 or 5T were not compared.
